# Relationality, Responsibility and Reciprocity: Cultivating Indigenous Food Sovereignty within Urban Environments

**DOI:** 10.3390/nu14091737

**Published:** 2022-04-22

**Authors:** Elisabeth Miltenburg, Hannah Tait Neufeld, Kim Anderson

**Affiliations:** 1 School of Public Health Sciences, University of Waterloo, 200 University Avenue W, Waterloo, ON N2L 3G1, Canada; emiltenburg@uwaterloo.ca; 2 Department of Family Relations & Applied Nutrition, University of Guelph, Guelph, ON N1G 2W1, Canada; kimberle@uoguelph.ca

**Keywords:** Indigenous food sovereignty, Indigenous food systems, Indigenous foodways, urban Indigenous Peoples, southern Ontario

## Abstract

There are collective movements of Indigenous food sovereignty (IFS) initiatives taking up place and space within urban environments across the Grand River Territory, within southern Ontario, Canada. Indigenous Peoples living within urban centres are often displaced from their home territories and are seeking opportunities to reconnect with culture and identity through Land and food. This research was guided by Indigenous research methodologies and applied community-based participatory research to highlight experiences from seven Indigenous community members engaged in IFS programming and practice. Thematic analysis revealed four inter-related themes illustrated by a conceptual model: Land-based knowledge and relationships; Land and food-based practices; relational principles; and place. Participants engaged in five Land and food-based practices (seed saving; growing and gathering food; hunting and fishing; processing and preserving food; and sharing and distributing), guided by three relational principles (responsibility, relationality, and reciprocity), framed by the social and physical environments of the place. Key findings revealed that employing self-determined processes to grow, harvest, and share food among the Indigenous community provide pathways towards IFS. This study is the first to explore urban IFS initiatives within this region, offering a novel understanding of how these initiatives are taking shape within urban environments.

## 1. Introduction

Indigenous Food Sovereignty (IFS) has been a living reality for Indigenous Peoples across Turtle Island who have sustained highly localized food systems since time immemorial [1,2]. IFS recognizes the sacred, participatory relationships between Indigenous Peoples and their foods, concretizing practices and knowledges that are shared to support Indigenous food systems [1]. Indigenous food systems are rich in ecological place-based knowledge, reflecting significant relationships to the Landscape and have evolved distinctly within communities over generations [2,3,4]. IFS is an action-oriented approach that supports these place-based food systems, through activities that consider the sociocultural meanings, acquisition, and processing techniques of Indigenous foods such as hunting, fishing, trapping, and foraging [3,5,6,7]. These activities are embedded within processes that support Indigenous knowledges, connections to Land (Throughout this paper Land is capitalized to acknowledge the personhood and sovereignty of Land as a Nation within the context of Indigenous governance and self-determination [8]), well-being and self-determination [9,10,11].

Indigenous Food Sovereignty has gained momentum in the last decade as Indigenous communities work towards revitalizing Indigenous foodways [1,10]. However, the uptake of these initiatives and associated research has predominantly occurred among northern and remote Indigenous communities within Canada. In these contexts, culture-based camps or on-the-Land programs are common pathways to strengthen IFS [10,12,13,14]. Gardening projects with associated food literacy programs or preservation workshops are more common in southern Indigenous communities to strengthen Land-based connections and revitalize Indigenous knowledges and skills [4,8,15]. This existing body of research offers important insights into how communities are implementing initiatives that can promote IFS, but findings are contextual to these spaces and places, with limited examples documenting community-based IFS initiatives within urban environments. 

There are a myriad of initiatives working towards IFS, reflective of the knowledge, food skills and relationship to Land held within each community. The published literature that explores ways to promote IFS within urban environments primarily consists of food skill workshops and establishing Indigenous gardens [11,16,17]. These initiatives are significant for urban Indigenous people to share knowledges, employ self-determination, and reclaim identity by practicing these skills and their cultures in the city [11,16,17]. Yet, there has been little research to-date that has examined the geographies and social contexts of urban environments [18,19]. This study aimed to address these gaps to better understand how urban environments influence IFS initiatives.

### Urban Indigenous Food Environments

The prevalence of food insecurity among Indigenous Peoples within Canada is a direct result of colonial policies and structures that have had detrimental impacts on Indigenous food systems [1,20,21,22]. Intergenerational impacts of colonial policies have disconnected Indigenous Peoples living in urban centres from the Land, shaping health disparities in distinct ways [18]. Indigenous Peoples living in cities face distinct impacts to accessing and engaging in Indigenous food systems when compared to their rural, remote, and on-reserve counterparts [23,24]. Research suggests that urban Indigenous Peoples are frequently unable to fully participate in Indigenous food systems and local food sharing practices because they are dispossessed from their physical and social environments [18,25,26]. As such, they may have divergent relationships to the Land and skills necessary to acquire and process or prepare Indigenous foods [11]. Urbanization is also a challenge to accessibility of Indigenous foods as market-based foods are more readily available in the local environment [26]. The majority relies on the industrial food chain in urban centres even though food can be accessed through alternative food networks, which include local farmers, hunters, and gatherers [27]. These overlapping systems make up the pathways representing how Indigenous food sources are accessed within the city, often relying on social and cultural connections to home and other Indigenous networks [18,25,26,27].

Indigenous Peoples within Canada are becoming increasingly urbanized, migrating to urban centres more than any other segment of the population [28]. Within the province of Ontario, 85.5 per cent of First Nations, Métis, and Inuit live outside of their home communities [28,29]. While 60 per cent of first and second generation urban Indigenous people retain connections, there are many who have been living in cities for two or three generations and are disconnected from their Indigenous foods and practices [23,30]. At the same time, urban Indigenous families would prefer to consume Indigenous foods more often due to their connections to wellness, Land, and culture; however, demand for these nutrient dense foods is often unmet in southern and urban settings [11,18].

Despite the growing numbers of Indigenous Peoples living within these diverse urban environments, there are few studies that consider the experiences of Indigenous Peoples in southern regions and cities where less visible circumstances of food insecurity are occurring [23,24]. A case study of the Indigenous Food and Medicine Garden at Western University highlights how growing traditional foods and medicines on campus supports Indigenous self-determination and Land reclamation but recognizes the institutional constraints that limit the potential of this IFS initiative [17]. The COVID-19 pandemic has simultaneously heightened these challenges in a range of environments [31], particularly in cities where an emerging number of Indigenous-led grassroots efforts are occurring [32,33] and becoming places of Indigenous resurgence [34]. This study highlights the Indigenous-led, community-based food sovereignty initiatives taking place in four urban centres within southern Ontario, Canada. The research presented in this paper is part of a larger study [35] that examined how the urban environment impacts local IFS initiatives. This paper focuses on findings specific to the social dimension of urban food environments, highlighting principles and practices that are components of IFS initiatives.

## 2. Materials and Methods

### 2.1. Community Context

The study took place within Waterloo and Wellington Regions in southern Ontario. These neighbouring regions include four major urban centres: Kitchener-Waterloo, Guelph, and Cambridge, all connected by the Grand River or its tributaries [36]. These cities have a total population of 761,391 according to 2020 census data [37]. It is estimated the region is home to 19,285 self-declared Indigenous Peoples, including First Nations (14,730), Métis (4585) and Inuit (480), together representing 1.5% of the total population [38,39]. The region has seen an increase in urban IFS initiatives within recent years in response to interest within the Indigenous community to reconnect with cultural foodways and support food security. Indigenous-led initiatives have emerged in connections to post-secondary institutions, Indigenous organizations, and grassroots community efforts. This study is embedded within a broader Canadian Institutes for Health Research (CIHR) funded research project which aims to bridge spaces and places between on-reserve, urban and educational environments to strengthen pathways towards Indigenous food sovereignty across southern Ontario.

Four existing IFS initiatives within this region were identified during the data collection period that began in the summer of 2020. Relationships were first established with Wisahkotewinowak, an Indigenous garden collective that maintained four garden sites across the region [40]. At the time, the collective provided Land-based learning opportunities for high school and post-secondary students. The foods and medicines harvested from the gardens were shared with post-secondary Indigenous student centres locally, through food preserving workshops, and a food share program in partnership with a local Indigenous agency. The Waterloo Region Indigenous Food Sovereignty Collective was also included as it had developed a neighbourhood approach, which began in response to the food insecurity needs at the onset of the pandemic [41]. The Waterloo Region collective had 18 gardens that ranged from an acre to individual backyard gardens. Shatitsirótha’, the University of Waterloo Indigenous Student Centre, was also involved and maintained a small produce garden, medicine gardens, and a ceremonial fire site [42], along with the North End Harvest Market in Guelph, which provides access to free produce and culturally significant foods to the local Indigenous community.

### 2.2. Theoretical Frameworks and Study Design

Indigenous research methodologies guided the study process by approaching the research through continual reflexivity of relationships, actions, and experiences to prioritize Indigenous ways of knowing [43,44,45]. These processes were applied by building relationships with Indigenous community members to centre participant voices and maintain responsibilities to those relationships [46,47]. Community-based participatory research (CBPR) methodology was applied to the study design as it is fundamentally driven by relationships and decolonizing power differences [48]. CBPR is a recommended approach when working with Indigenous communities [46,47,49]. The participatory elements of CBPR supported the relational, action-oriented nature of IFS [1]. The participatory nature of IFS provided opportunities for the first author to build relationships and develop a research inquiry that could be useful, accessible and give voice to the community members leading IFS initiatives in these urban contexts.

Participating in IFS initiatives was also an important aspect of reducing power differentials between the lead researcher and the community. Particularly, the lead author of this study (E.M.) is a non-Indigenous researcher who moved to the region at the start of her MSc program. She relied on the second (H.T.N.) and third author (K.A.) to support her gaining trust and reliability within the community, as they both live and work with Indigenous communities in the region. By relying on these pre-existing relationships, the lead author was able to develop independent relationships with community members and connect with other IFS initiatives in the area. By working alongside and learning with Indigenous peoples and their initiatives, she was able to combine the knowledge gained through the research and relationship building on the Land into useful results and recommendations.

CBPR allows for research with Indigenous communities to be validated by following the OCAP^®^ principles of ownership, control, access, and possession of knowledge [49,50]. The USAI Research Framework also guided the research process and design [51]. The study applied the principles of utility, self-voicing, access, and inter-relationality through each phase of the research. A research advisory committee was established to bring together community partners to advise research activities relating to the urban component of the CIHR grant. This group included representatives from Indigenous student centres on post-secondary campuses, leadership from local Indigenous organizations, community members engaged in IFS initiatives, as well as other research faculty and students. A few members of the committee were recruited as research participants, and other research participants were invited to join the meetings. As the formal research process unfolded, research updates and findings were continuously shared with community members and research participants at research advisory meetings to ensure knowledge presented was accurate and authentic. Research participants and community members had an active voice in contributing to the development of the research. These relational processes were integral for this research to be valid, useful, and relevant to those individuals and networks leading the local community-based IFS initiatives.

### 2.3. Participant Recruitment

Purposive sampling was utilized to recruit participants through relationships established with the Wisahkotewinowak Collective, to then include other organizations and initiatives. The community-based aspect of this research helped identify participants who were engaged in local IFS initiatives, self-identified as Indigenous and lived in the urban centres of Kitchener-Waterloo, Guelph, or Cambridge, Ontario. Snowball sampling was then used to build the sample through local networks [52], drawing on the relationships participants held with others in the community. Once invited formally via email, participants were offered asemma (tobacco) to affirm the intentionality of the research purpose [53] and respect cultural protocols acknowledged by the local Indigenous community. Recruitment and data collection occurred between August and November 2020. Ethical approval was received from the University of Guelph Research Ethics Board (REB#20-02-021). 

Seven participants were recruited to participate in the study. At the time of recruitment, five participants were living in Kitchener-Waterloo, one in Cambridge and one in Guelph. Two participants identified as male, four identified as female, and one identified as Two-Spirit. Two of the participants were youth (under 30), four were adults (31–64), and one participant was Elder (over 65). Participants were given the option to be identified by their name or a pseudonym. Participant characteristics are included in Table 1.

### 2.4. Data Collection and Analysis

A semi-structured interview guide was developed in collaboration with the research advisory committee to explore participants’ perspectives of Indigenous foods, engagement in urban IFS initiatives, impacts of the urban setting as well as gather their recommendations to strengthen this work in the future. The interviews lasted between one hour and two hours in length. Interviews were held either in-person or virtually through a secure video conferencing platform. In-person interviews were conducted outdoors at a safe physical distance to satisfy COVID-19 protocols at the time. Interviews were recorded with the consent of the interviewee. One participant chose not to be recorded, instead, they typed out their responses to the list of interview questions after conversations were shared discussing the study concepts. This document was treated as the transcript for data analysis. Audio-recorded interviews were transcribed verbatim by the lead author, with support from an undergraduate student. Individual transcripts and quotations incorporated into the results were shared with participants as a form of member checking [53], allowing participants to edit quotations so their words were accurately reflected, and any unwanted identifiable details could be removed. At the request of participants, additional slight edits were made to their quotes to improve readability.

The lead author completed a thematic analysis of the transcripts, where emerging themes and concepts were highlighted for further exploration. Codes were then highlighted and organized using NVivo 12 software. A combination of deductive and inductive coding was applied to identify themes in the data. Coding was complete once codes captured the patterns and outliers within the data [52]. Preliminary themes were shared with the research advisory committee and interested participants for feedback before results were finalized. These processes associated with the previously identified methodologies strengthened the overall research by prioritizing Indigenous knowledge and centring participant voices [48,54].

## 3. Results

**Figure 1:**
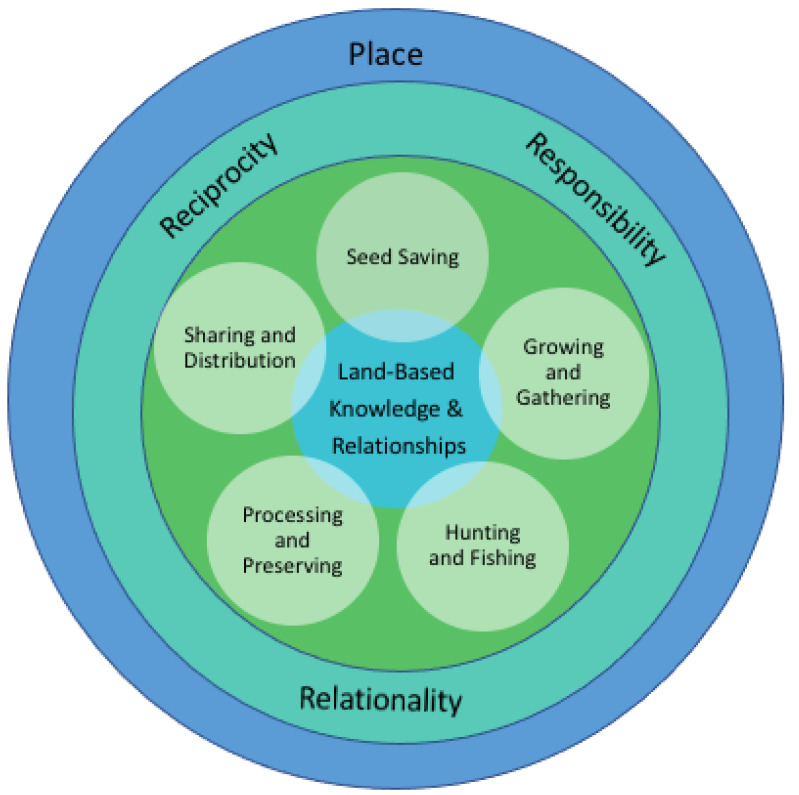
Conceptualizing urban Indigenous food sovereignty initiatives in Grand River Territory.

Thematic analysis revealed four interrelated themes: Land-based knowledge and relationships; Land and food-based practices; relational principles; and place. Participants engaged in five Land and food-based practices: (1) seed saving, (2) growing and gathering food, (3) hunting and fishing, (4) processing and preserving food and (5) sharing and distributing food. These practices centred around Land-based knowledge and relationships, guided by principles of responsibility, relationality, and reciprocity. The relatedness of these themes are presented in a conceptual model (see Figure 1). This model illustrates components of urban IFS initiatives, which are influenced by place. Place is situated in the outer layer of the model, which contextualizes the social and physical aspects of the urban environments that make up the Grand River Territory. The findings presented in this paper highlight how the three principles were evident in Land and food-based practices, suggesting these principles and practices construct the *processes* that are integral to urban IFS initiatives.

### 3.1. Relationality, Responsibility and Reciprocity

When describing engagement in urban IFS initiatives, all participants spoke about relationships to Land, seeds, plants, food, and people in the community, demonstrating that relationships were integral throughout IFS initiatives. Participants enacted these relationships by being relational, responsible, and reciprocal in the ways they carried out Land- and food-based practices. These themes were categorized as relational principles that surround Land- and food-based practices as illustrated in the model, simultaneously supporting and interacting with each other. Relationality encourages responsibility, which requires reciprocity.

All participants spoke about being relational when carrying out Land and food-based practices. These conversations stemmed from the way participants described their personal relationships with food. Interviews contained discussions around definitions or interpretations of Indigenous or traditional foods. It became apparent that what connected participants to these foods was their relationships. Sarina spoke to the emotional significance of Indigenous foods, comparing the feeling to the love you have for a sibling. She stated: “I feel like that emotion is mirrored in a relationship with food because it is such a large part of how you are supported-because you wouldn’t be able to live without the foods.” Beth spoke about how there is a spiritual significance to food grown and shared in community. She explained, “there’s a ceremony to it. Right? There’s a thankfulness. There’s restraint versus [referring to market foods] just-it’s on sale I’ll take the whole shelf.” These illustrations demonstrate there is a sacred and emotional relationship to Indigenous foods.

This relational understanding of food extends to how people work together in community to support IFS initiatives. Rachel recognized how IFS initiatives require participation for many people to come together and do the work, strengthening social networks. She commented, “now that there’s more community involved there’s some people that have made new friends or made new connections and have more people to reach out to.” Therefore, being relational within these contexts appears to be a core principle of urban IFS initiatives and builds community.

Five participants spoke about how being relational comes by knowing one’s responsibilities. Participants reflected on their own teachings about their role as a human being in Creation and having a responsibility to the Land. For example, Nookomis talked about knowing how to be “responsible,” pointing out that acting or living responsibly begins with the Anishinaabe Creation Story:

*Creation ensured that all created holds a part of Creation/Spirit. It might be said that this “holding of Spirit” was the first commitment/responsibility/treaty between Creator and that Created…This responsibility honours the reciprocity, how we give back to Creation’s Garden, Mother Earth.*

Responsibility to Land is made possible in urban centres by participating in IFS initiatives, which foster relationship-making with all Creation. For instance, Beth explained how she understands her responsibility for the Land around her. She summed up: “Well because I’m on it. And my feet are on it. So, where Creator has my feet, there I will be responsible.” Therefore, being responsible for the Land does not matter where you are physically located; rather, it’s about how you relate to the Land you are on.

Reciprocity also emerged as a value participants carried throughout their engagement in IFS initiatives. Reciprocity was required to uphold responsibilities and support relationships. Two participants spoke directly about how reciprocity was enacted through local food sharing, in response to gifts provided from Land and Creation. Sarina reflected the reciprocal aspects of food sharing activities:

*It’s understanding I have much more than I need, and I have the capacity to share with others, so I need to do that in order to satisfy my responsibility to the Land and others around me…I see it as connecting to our sense of responsibility that is linked to reciprocity. Inherently, we have a responsibility to the Land and that is to first and foremost harvest in a respectful way. But then I think that responsibility extends to the way we share the food.*

In this quote, Sarina speaks to how reciprocity fulfills responsibility, and strengthens relationship–not just to people when food is shared with but also with the Land. Nookomis extended this idea, noting that reciprocity is inherently linked to honouring responsibility to Creation, with humility and gratitude:

*As Creator knows me, I act on my original instructions. I walk this red path in the physical realm, to observe, listen and learn from seeds, plants and all those other Relations, our Kin. We are all interconnected. One of the ways I honour with humility and gratitude, this Earth our Mother and All Ancestors, is by being a seed keeper of a number of plants/roots/barks/flowers/seeds specific to my Clan. I do this in Ceremony, with Community to produce, gather, prepare, feast and for healing. I do this for now and for the generations of All Our Relations whose faces we have yet to see.*

Reciprocity places a sense of accountability on the responsibilities and relationships to Land and community. Creation provides gifts that can be reciprocated back to the Land by sharing food, knowledge, and ceremony with others in community, who also belong to the Land. Reciprocity is an important value displayed within urban IFS initiatives, as it ensures a continual cycle of giving and receiving that is generated from a place of humility and generosity. This cycle of reciprocity, relationality and responsibility guided Land and food-based practices.

### 3.2. Land- and Food-Based Practices

Participants in this study spoke about five Land- and food-based practices that were core components of local IFS initiatives. Six participants discussed the significance of seeds and seed saving as a practice. Rachel explained that part of her group’s efforts was designated solely for seed saving, which was growing enough seed to share with community next year. Beth explained how planting and saving seeds are essential to initiate and continue Indigenous food sovereignty efforts:

*It starts with seeds, right? Every person having seeds in their hand and those seeds growing food for themselves, their families, their community, but also being able to put some aside for seeds again. [There’s] that cycle of provision; giving and preparation. Provision for your family, for your neighbour, giving of seeds back to community for others who don’t have what they need yet.*

Participants shared how seed saving was about honouring relationships and responsibilities. For instance, Garrison shared the importance of growing and saving succotash beans and Lenape squash to connect with his identity from his ancestral Territory. Beth recognized the significance of growing white corn from Six Nations in the urban garden spaces, which are situated on Haudenosaunee Territory. She grew this corn out of respect for Haudenosaunee people, explaining, “because of the granting of the Land in this Territory to them, which we all know has not been fulfilled, but just doing my best to honour their traditions and their seeds on the Land.” She continued and explained that despite not being Haudenosaunee herself, making sure these seeds are in the ground is a way to show mutual respect for her Indigenous sisters in community.

Growing food was the most common practice to acquire Indigenous foods and medicines in the city, as all the local initiatives discussed by the participants involved gardens. Four participants also spoke about gathering food by foraging and two more about gathering sap from maple trees within the city limits. Participants also described how their gardens were used to grow for different purposes, including for seed, medicines, Indigenous foods, or conventional garden produce. Several participants recognized the importance of cultivating culturally significant foods in urban spaces to strengthen relationships to the Land they were growing on and honour the responsibility to Indigenous ancestors who cultivated these plant varieties over thousands of years. A list of these Indigenous plants described specifically by Garrison, Dave, and Lori are listed in Table 2. Garrison described how growing plants and saving seeds supported the relationship he held to Land while also upholding responsibilities:

*Planting and saving seeds and planting again are these beautiful ways of really being able to witness the unfolding aspect of life. So, I was always really intrigued by that and really excited to be able to use my hands to co-create with, in relationship with these plants, but also in relationship with the Creator and what the Creator had intended for us to be, right? Cause these relationship beings, when we receive from those plants that we are also, that our responsibility is to save those seeds and keep that plant alive.*

Six participants spoke about hunting or fishing when describing access to culturally significant foods around IFS initiatives. Three were involved in hunting and fishing practices locally, but only Dave spoke about fishing within an urban area for personal consumption. None of the participants mentioned hunting or fishing as activities that took place within their IFS initiatives, suggesting limited access to these foods within urban settings. Rachel described how she would like to increase opportunities for hunting and fishing within the region noting, “we don’t have very many hunters right now, but we are hoping to teach some more so we are basically mentoring those who don’t know.”

Three others had personal connections to family or community that supported access to wild meat and fish. Beth mentioned, “My Dad is not First Nations, but he was a hunter and so we would eat game. Like he would fish and hunt so we would have wild food from that.” This suggests the importance of relationships as well as connections to knowledge and practice that are necessary to access game locally while living within urban environments. This aspect of relationality was similarly emphasized by Lori, who noted the difference between her social networks to access wild meat after relocating to Ontario:

*Back in Saskatchewan when I was in an urban setting, I had a little bit more access to* [wild meat] *because I had a network of people. And so, it was still relatively easy to get or to trade for wild meat… but when I got out here, I didn’t have that network and I still don’t really have strong enough ties* [here] *to access wild meat.*

Another practice participants described was processing and preserving foods. Five participants mentioned how they processed or preserved locally grown produce to create more variety in food availability and extend the growing season. Dave and Lori had connections to Indigenous student centres on campus, so foods harvested from the gardens supported food-based programming for Indigenous students. For Lori, this included soup and bannock days and the fall feast. Staff and students would prepare the food for these events. Beth and Rachel talked about how they relied on people in the community with canning skills to support food processing and preserving efforts. Rachel described how the community helped forage and harvest fruits and vegetables from their backyards and city spaces to then be processed and preserved. She explained, “we’ve also been collecting equipment, so we have five pressure canners for the canners that are community canners.” This sharing of equipment and responsibilities demonstrates how individuals in the community can support IFS initiatives by participating in activities where they can best lend their resources, skills, labour, and time.

All participants spoke about sharing. Food sharing happened through organizational distribution avenues, as well as more informally, through personal networks. Dave and Rachel talked about how food distribution pathways were created within their IFS work in response to the COVID-19 pandemic to increase access points and feed the local urban Indigenous community. Dave spoke about an Indigenous food basket sharing program that began with a partnering Indigenous organization, recalling, “we did our first official give away of food and did thirteen boxes of produce to Indigenous community members.” Similarly, Rachel expressed, “in the beginning we had about 30 people we were feeding regularly [with] either a weekly grocery bin of different foods to make themselves or meals made every day.” An unexpected result that Rachel shared was that she would receive kind messages from members of the urban Indigenous community who were not only in need of food, but social connection. Lori also reflected on how food was a way to support relationships in the community, by making and sharing food to help people stay connected. Sarina spoke to these broader impacts:

*I think that our work has not only been able to nourish bodies in terms of nutrients but also souls and spirits of people. Not everybody that we’ve been giving boxes to are in dire, dire need of food physically. But they may be kind of spiritually deprived because they are stuck at home doing Zoom calls by themselves.*

Rachel described how sharing was inherent throughout Land and food-based practices, stating, “we have seed sharing that’s been happening, food growing, meal making and food sharing.” The practice of sharing was an important way to build relationships and uphold responsibilities to community in reciprocal ways. For instance, Beth stated, “It’s looking at what I have, what I’m responsible for, as my community’s not mine. And sharing my time, my resources that I’m responsible for with my neighbours. My brothers and sisters in community.” Sharing strengthened relationships in response to fulfilling responsibilities. Sarina described these interactions, demonstrating how relationships are central to IFS initiatives:

*We kind of operate in a circular way, like we’re all equal… and that’s translated to how we share our food… And I think that there’s something refreshing but also beautiful about it because in each exchange between people throughout the process of food growing and food giving there’s a reciprocal thing going on that builds relationship…because the entire process has to do with community really, and relationship with others. Not only do we rely on volunteers, and the combined collective work of all of us, but we also rely on the Earth and the Land to teach us and help us through the process.*

Sharing food was not simply a practice, but a significant process that unified applications of responsibility, relationality, and reciprocity within IFS initiatives, strengthening relationships between Land, the natural environment, and the social networks within community. The model conceptualizes urban IFS initiatives across Grand River Territory, but it also represents this web of relationships between all elements within the environment. This idea is noted by Nookomis who shared, “Creation is the system….it is holistic and sustainable; it is our identity. There are no separations between two leggeds and all else created. We are of the Land.” This continual thread weaves through each Land- and food-based practice, connecting these actions as components of a more holistic system, clearly governed by a set of principles that are really at the heart of IFS initiatives: being relational, responsible, and reciprocal. 

## 4. Discussion

Overall, the results of this exploratory study offer an understanding of community-based IFS initiatives within urban environments. The participants in this study demonstrated that relationality, responsibility, and reciprocity are core values of urban IFS initiatives, which guided Land- and food-based practices. The model demonstrates how IFS initiatives are interdependent: a connection to Land is essential for food to be grown, and growing food to share with community fulfills a responsibility to the Land. Participating in Land and food-based practices connects people to their responsibilities by saving seeds, growing, processing, and sharing food in relational and reciprocal ways. This model conceptualizes an urban Indigenous food system within Grand River Territory, identifying key processes that work towards IFS. This discussion section engages with principles of IFS and describes how relationality, responsibility, and reciprocity are demonstrated within community-based IFS initiatives. These processes can be applied to other urban Indigenous communities, offering a pathway towards strengthening IFS.

The results from this study concur with other research on IFS and bring new understandings of IFS within the urban context. Participants in this study identified that Indigenous foods have a sacred aspect to them because of the relationship one has with their food. This parallels with findings from another urban-based study in Winnipeg, Manitoba where urban Indigenous Peoples recognized the relationship with food as a spiritual process; the growing, harvesting, preparing, eating, and sharing cultural food was an act of ceremony [11]. The spiritual significance of Indigenous foods has been well documented in the literature [4,8,11,26,55,56], but this study adds how urban Indigenous Peoples are enacting the IFS principle of food as sacred through urban initiatives. The sacred, emotional, and relational significance of Indigenous foods was recognized by participants in their understanding of responsibilities stemming from the innate relationship with Land or Creator. This was also acknowledged by Robin [9] who states: “relationships to the Land are also important in helping to fulfil the roles and responsibilities we have made towards the Land as caretakers” (p. 92). Responsibilities were fulfilled by active participation in Land and food-based practices through the planting, harvesting, processing, and sharing food with others in community as has been identified by Michnik, Thompson, and Beardy [13]. Their study examined a northern food initiative at Garden Hill First Nation and described the sacredness of food requires active participation in Land-based activities. Similarly, in the southern community of Kahnawà:ke, Delormier and colleagues [4] have highlighted how Haudenosaunee food sovereignty focuses on restoring Indigenous cultural responsibilities and relationships to Land, each other, and the natural world. This research aligns with the findings from our study as participants honoured the sacredness of food by displaying responsibility and participating in IFS initiatives.

Participants in this study upheld responsibilities to Land and community primarily through the sharing of food as an act of reciprocity, recognizing the gift of food that comes from the Land. Cidro and colleagues [11] also describe the connection that cultural foods provided to the Land through reciprocity within the city of Winnipeg, Manitoba. Participants described that they could still be connected to the Land through their relations who gifted and shared food, despite not always having access to Land or being able to engage in food-based activities. This demonstrates that reciprocal relationships extend beyond the people who harvested food and highlights, “the larger process of food giving up its life to support people” [11] (p. 36). These findings are consistent with results presented in this paper, confirming that relationships are the mitigating factor that links people to Land and place in urban settings—and reciprocity ensures the sacredness of these relationships continues. The sacred gift of food connects urban Indigenous Peoples to Land and community, unifying the spiritual relations in the local environment.

The relational principles that guide Land- and food-based practices support processes of self-determination. In this study, the urban Indigenous community collectively demonstrated acts of self-determination by saving seeds and deciding what foods and plant medicines to grow. Participants chose to cultivate foods they held personal connections to, and also grew varieties that held local significance for the Anishinaabe and Haudenosaunee peoples, recognizing the Treaty Territory that they currently live in. These self-governing practices are what Daigle [5] acknowledges as “central to this larger process of decolonization and self-determination” (p. 13). The decision-making processes and social systems are pathways to self-determination that can ensure sustainable food systems [4]. Participants in this study similarly demonstrated self-determination through establishing processes to harvest, preserve, and distribute food within the community. These food sharing practices valued food as a gift–not a commodity to be exchanged transactionally.

Sharing food was a significant Land- and food-based practice, but also a process that enabled participants to be relational, responsible, and reciprocal in community-based IFS initiatives. In this study, sharing food supported social relations and access to culturally significant foods to the wider Indigenous community. Previous research shows how sharing and trading promotes community and social cohesion while also increasing access to culturally significant foods for Indigenous Peoples in the city [11,18,23,24,26]. The Indigenous community in the city of Saskatoon recognized that preparing and eating food within kinship networks contributes to resistance to the dominant insecure food system [27]. Therefore, implementing food sharing practices offers an alternative approach for urban Indigenous Peoples to determine their food systems.

Sharing food is embedded within Anishinaabe and Haudenosaunee food systems and practices, so enacting these processes in the urban centres supports self-determination and strengthens community relations. Growing food supports IFS through very tangible ways of increasing food availability, but implementing processes and systems that govern how food is distributed and shared within community promote self-determination, foster reciprocity and collective citizenship [57]. Gardening initiatives can increase community control over food produced, which can support a sharing economy that meets community needs [13]. Enacting IFS initiatives within urban environments prioritizes responsibilities over rights, as exhibited in this study by participants taking care of each other and the Land. Timler and Sandy [58] offer that shifting our understanding of food and Land from one of resource to one of relationship creates the opportunity to rebuild relationships through reciprocity among neighbours in responsible ways (p. 18). Perhaps, implementing IFS initiatives that centre relationality, responsibility, and reciprocity provides a pathway towards self-determined food systems within urban environments. 

IFS initiatives strengthen Indigenous foodways that support local food insecurity needs [25], while building community cohesion, and wellness [58]. Self-determination over Indigenous foods and food systems contributes to community wellbeing through sharing food practices, ceremony, and social gatherings [59,60]. Recent findings from a Syilx-led IFS initiative that revitalized Sockeye salmon in the Okanagan River Basin of British Columbia resulted in enhanced cultural connectedness, increased a sense of belonging and wellbeing [61]. Indigenous food systems embody healthy processes and acquisition to nutritionally and culturally significant foods [6,26,56]. Previous research has established the importance of community to foster relationships with Land and carry out food-based practices, which are inherently collective activities [11,18,62,63]. As Pawlowska-Mainville [63] state, “food sovereignty needs a community to feed a community” (p. 63). Participants in the present study demonstrated how community was in essence created by engaging in IFS initiatives. Sharing not only of food, but seeds, knowledge, and resources, are key components of urban IFS initiatives that enabled participants to be relational, responsible, and reciprocal in self-determining ways. Therefore, IFS initiatives can create supportive social environments in the urban context resulting in physiologically, spiritually, and emotionally healthy people and communities.

### Recommendations for Future Research

This research highlights how IFS initiatives are taking shape in urban environments within southern Ontario, providing a novel understanding of how urban Indigenous Peoples are pursuing food sovereignty initiatives. Further research is, however, necessary to understand how the principles and practices of other urban Indigenous communities compare to the findings presented in this study. Communities must define what approaches work best for them because of the diverse socio-historical circumstances that affect local experiences of IFS [5,9,62,64]. Indigenous food systems involve governance structures based on the ethics of reciprocity, sharing and valuing food as a sacred gift [2,63,65]; therefore, further research could explore how food economies based on sharing, harvesting, and gifting may provide alternative approaches that increase food security, food access, and support the decolonizing goals of IFS within urban environments.

At the local and community levels, there are opportunities to bring Indigenous Peoples and settler food actors into dialogue for solidarity building [5] and equity within local and regional place-based food systems [66]. Bringing together key actors within the community can be completed through a collaborative, cross-sector governance model, network, or space that can work towards policies that support self-determination within Indigenous food systems and practices. The Indigenous Food Circle based out of Thunder Bay [67] provides an example of the unique partnership described between the local food policy council, health unit, Indigenous communities, and post-secondary institution. At the same time, Indigenous women must be centred in these spaces because Indigenous leadership that follows a matriarchal system of governance is a move towards self-determination [9,68]. There is a gendered element to Indigenous food sovereignty work not examined in this study, warranting direction for further research.

## 5. Strengths and Limitations

This study is the first to document the growing movement of urban IFS initiatives taking place in Grand River Territory, highlighting how urban Indigenous Peoples are engaging in Land- and food-based practices in self-determined ways. A strength of this study is the application of CBPR and relational accountability built within the Wisahkotewinowak Collective and the wider community-based work taking place in the region. Through the participatory, community-based nature of this study, results supported and strengthened the innovation around IFS, pointing towards ways this work can expand to other urban IFS movements. This research also highlights opportunities for non-Indigenous people to participate in and learn from Indigenous community members leading food sovereignty work to create sustainable and supportive environments in urban contexts.

The location and food availability are important to acknowledge as the local context of this study limits the generalizability of the research findings to other southern, urban contexts. Growing food is a common IFS practice within southern Ontario because of its ideal climate and arable land. However, other urban contexts may reflect different IFS practices, depending on the local food availability and other environmental factors. Another limitation of this study was implementing community-based research and data collection with human participants during the onset of a global pandemic. COVID-19 limited in-person gatherings and opportunities to network and recruit participants through community events. This resulted in a smaller sample size than anticipated. This also challenged original data collection and dissemination plans, delaying timelines and having to adjust methods that complied with ethics approval. Instead of holding in-person interviews, video conferencing was prioritized and a knowledge sharing presentation was held virtually.

## 6. Conclusions

Indigenous food initiatives continue growing at the grassroots level across Turtle Island. Despite this, there is limited research that explores IFS initiatives in urbanized centres within Canada. The research provides novel insights to the body of the IFS literature by considering how the urban environment impacts IFS initiatives. By examining components of IFS initiatives, this study offers considerations for what constitutes Indigenous food systems in the urban setting. The core values of relationality, responsibility and reciprocity demonstrate how social relationships can be structured to carry out Land- and food-based practices to promote self-determination and increase community wellbeing. These results also provide insight into ways that both Indigenous and non-Indigenous people living in a common place can come into relationships to cultivate care for the Land and the community by participating in urban IFS initiatives. Growing, harvesting, and sharing food together can help foster a respectful way of living that honours Treaty responsibilities and supports the sustainability of IFS initiatives into the future.

## Figures and Tables

**Table 1:** Interview participant characteristics (*n* = 7).

Name or Pseudonym	Location	Indigenous Identity	Connection to IFS Initiative
Dave	Kitchener	Métis	Wisahkotewinowak
Garrison	Kitchener	First Nations	Wisahkotewinowak
Sarina	Kitchener	Métis	Wisahkotewinowak
Beth	Cambridge	First Nations	Waterloo Region Indigenous Food Sovereignty Collective
Rachel	Kitchener	First Nations	Waterloo Region Indigenous Food Sovereignty Collective
Lori	Waterloo	Cree-Métis	Waterloo Indigenous Student Centre-Shatitsirótha
Nookomis	Guelph	First Nations	North End Harvest Market

**Table 2:** Examples of Indigenous foods cultivated by study participants for Indigenous food sovereignty initiatives.

Beans	Corn	Squash	Medicines	Berries
Cherokee Trail of Tears Succotash Red Runner	Lenape Blue Mohawk White	Arikara Lenape Gete Okosomin	Sweetgrass Tobacco Mullein Pearly Everlasting Mountain Sage Prairie Sage	Strawberries Saskatoon

## Data Availability

The data are not publicly available due to privacy.
